# Intrauterine ovarian dermoid cyst complicated by torsion: an uncommon presentation of abdominal mass in a neonate

**DOI:** 10.1259/bjrcr.20210137

**Published:** 2021-11-03

**Authors:** Darakhshan Kanwal, Safaa Khalil, Khaled Attia

**Affiliations:** 1 Department of Radiology, Al Qassimi Women and children Hospital, Sharjah, United Arab Emirates; 2 Department of Radiology, Al Kuwait Hospital, Sharjah, United Arab Emirates

## Abstract

Fetal ovarian cysts are the most common abdominal masses in the female fetuses and believed to be caused by *in utero* exposure of fetus to maternal and placental hormones. Majority of them are diagnosed in third trimester and should be distinguished from other causes of abdominal masses of genitourinary and gastrointestinal origin. Once diagnosed serial ultrasound monitoring is recommended to document changes in size or appearance. Complications like torsion or rupture merit careful assessment and surgical intervention to preserve ovarian function and fertility.

We report a case of intrauterine ovarian dermoid cyst complicated by torsion, which was diagnosed prenatally on ultrasound as complex cystic lesion within the abdomen.

## Case report

### Clinical presentation

An 8-day-old female child was brought to the clinic for work-up of cystic lesion in right lumbar region diagnosed on prenatal ultrasound. The child was full-term born at 38 weeks by normal vaginal delivery. Currently, she is on total breastfeeding. Her antenatal ultrasound showed a well-defined complex cystic lesion in the right lumbar region extending down to the urinary bladder with peripheral vascularity.

On arrival child was active, afebrile. Heart rate 133 /min, respiratory rate 42 /min, SpO2 99%, weight 2.9 kg. On examination, abdomen was soft, not distended. A mobile palpable mass in the right lumbar and iliac fossa region was noted about 5–6 cm. Normal anal opening, normal female genitalia were observed.

## Work-up

Laboratory examinations revealed hematological and biochemical test results within normal limits. Ultrasound revealed a well-defined right para midline cystic mass within the abdomen. It measured 5.2 × 4.3 x 3.5 cm. The cyst was thick walled and showed mesh like areas of reticulation. Hypoechoic rim and few follicles were noted at the periphery. Vascularity was detected in the wall. The mass was clearly separable from right kidney. Uterus and left ovary were unremarkable, right ovary was not visualized. Mild free fluid was also noted within the abdomen. Differentials diagnosis of complicated right ovarian hemorrhagic cyst with possibility of torsion was given.

## Management

Diagnostic laparoscopy with possible cystectomy ± right oophorectomy was planned. Laparoscopic examination revealed right ovarian chocolate cyst about 4 × 5 cm causing a twist for ipsilateral ovary with attached fallopian tube making three turns. The omentum was included within the twist. Left ovary and fallopian tube was normal. Surgical excision of torted right ovarian cyst with gangrenous ovary and fallopian tube was performed and sent for histopathology. The patient was discharged on third post-operative day in stable condition.

## Outcome

Serum alpha-fetoprotein levels were high, *i.e.* >830.00 U ml^−1^ (Normal range 0.0–6.72 U ml^−1^). Histopathological examination showed right ovarian cyst with gangrenous change consistent with torsion. The morphology was suggestive of dermoid cyst.

## Discussion

Fetal ovarian cysts are the most common abdominal masses diagnosed in female fetuses with reported incidence of about 1 in 2500 pregnancies.^1^ These cysts are typically diagnosed during the third trimester due to hormonal effect (*e.g.* maternal estrogen, fetal gonadotrophins and placental human chorionic gonadotrophin).^2^

Ultrasound criteria for diagnosis of ovarian cysts include^3^

1. Confirmation of female gender
2. Cystic structure that is regular in shape and located off the midline
3. Size ≥20 mm in diameter (to differentiate from maturing follicles)
4. Identification of normal urinary tract anatomy
5. Identification of normal gastrointestinal structures

Another criterion proposed by Nussbaum’s et al suggests that cysts can be categorized as “simple” or “complex” based on their sonographic appearance.^4^

1. Simple cysts that are completely anechoic on ultrasound.
2. Complex cysts can have an echogenic wall, internal septae, fluid-debris level, or a blood clot.

Once diagnosed it should be differentiated from enteric duplication cysts, lymphangiomas, renal cyst, multicystic dysplastic kidney, meconium pseudocyst, choledochal cysts, ureterocele and hydrocolpos in definitive diagnoses. The characteristics used to distinguish ovarian cysts from other cystic masses are summarized in Table 1.^5^

**Table 1.** Differential diagnosis cystic abdomino-pelvic mass in fetus

Type of mass	Imaging characteristics
Ovarian cyst	Female fetus only; “daughter cyst” sign (smaller cyst within larger ovarian cyst)
Enteric duplication cyst	“Gut signature” sign (cyst wall is thick and layered)
Lymphangiomas	Thin-walled multilocular cystic mass with septations; infiltrative; may involve the body wall
Renal cyst	Solitary unilocular cyst in the renal parenchyma; renal architecture preserved
Multicystic dysplastic kidney	Multiple macroscopic anechoic cysts that distort reniform shape; mass adjacent to the fetal vertebral column
Meconium pseudocyst	Irregular shape; may be thick walled; conforms to contours and liver surfaces
Choledochal cyst	Unilocular cyst that communicates with the bile ducts; located the right upper quadrant of the abdomen
Ureterocele	Thin-walled anechoic cystic mass in the bladder; often associated hydroureter and hydronephrosis
Hydrocolpos	Fluid-filled midline pelvic mass posterior to the bladder; may associated with uterine dilatation (hydrometrocolpos)

Although fetal ovarian cysts can cause fetal anemia, compression, or rupture during the antenatal period, the most common complication is intracystic hemorrhage, followed by torsion.^6^ Ovarian torsion results from either partial or complete twist of the ovary and fallopian tube as in our case where ovarian cyst caused a twist for ipsilateral ovary with attached fallopian tube and omentum. Two peaks for age distribution of pediatric ovarian torsion have been reported, one occurring in infants and the other occurring at 12 years of age.^7,8^

The primary goal of management is to preserve ovarian tissue however, it is controversial. Although approximately 50% of prenatally detected cysts regress spontaneously, 35% of them develop in association with complications such as ovarian torsion and hemorrhage.^9^ Several management options are described in the literature, including prenatal ultrasound monitoring, antenatal aspiration of simple cysts to prevent torsion and finally, observation or resection of all complex cysts in the neonatal period.^10^

Enríquez et al reported 11 cases of clinically asymptomatic complex ovarian cysts, which were managed conservatively and showed involution by the age of 1 year. Therefore, they suggested that surgical intervention should be pursued in cases of large complex cysts which are symptomatic or do not regress spontaneously.^11^ Similarly in a retrospective study done by Perez et al, it was found that fetal ovarian cysts is an isolated entity and surgical intervention was indicated in larger cysts (>4 cm) or when complications are suspected.^12^ In our case, indications for surgery were large size (>5 cm), presence of internal septations, echoes, thickened wall and absence of vascularity suggestive of torsion.

In a study done by Trinh et al, conservative approach was preferred, asymptomatic infants were followed-up clinically with ultrasound and surgery was performed only in complicated cases.^5^

## Learning points

1. Fetal ovarian cysts are reported as most common abdominal masses in female fetuses, yet should be differentiated from other causes of genitourinary and gastrointestinal cystic masses.
2. Ultrasound is the preferred modality of choice in diagnosis and follow-up, as it is non-invasive and readily available.
3. Once diagnosed careful monitoring is mandatory to observe for risk of complications.
4. The primary goal of management is to preserve ovarian function and fertility; however, it is variable as most of them regress spontaneously but many develop complications.
5. Surgical intervention is only indicated in cases where complications are suspected.

**Figure 1.**
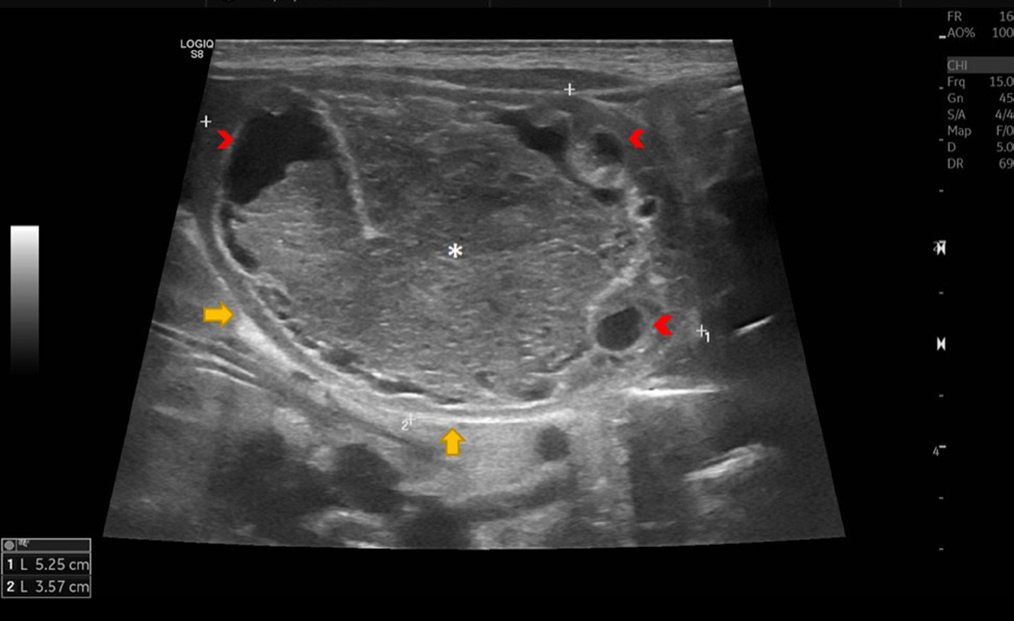
Well-defined right para midline thick walled cystic mass (block arrow) shows mesh like areas of reticulation (asterisk). Hypoechoic rim and few follicles at the periphery (arrowhead).

**Figure 2.**
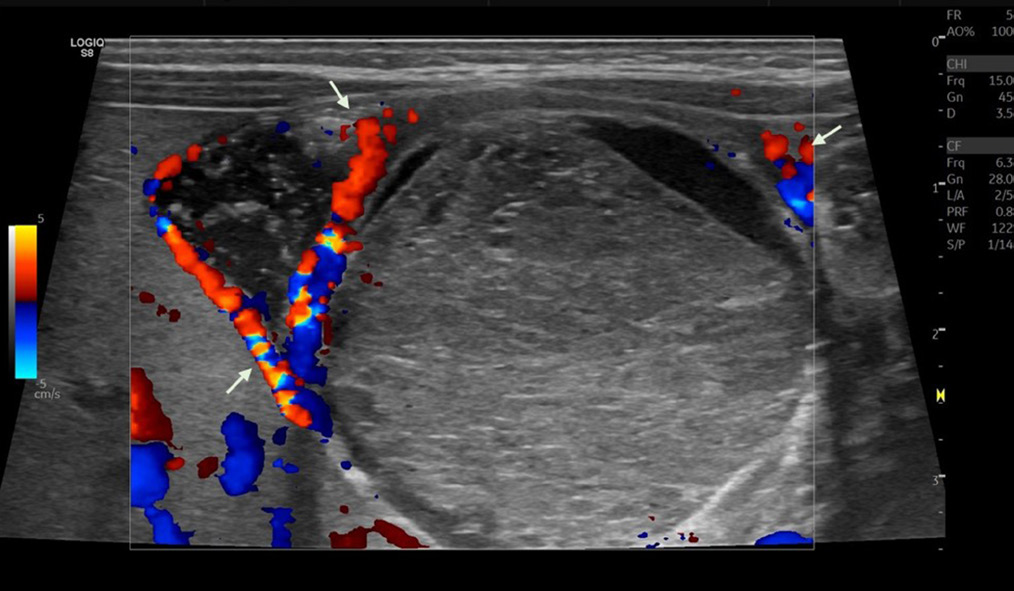
Vascularity detected at the periphery (arrow).

**Figure 3.**
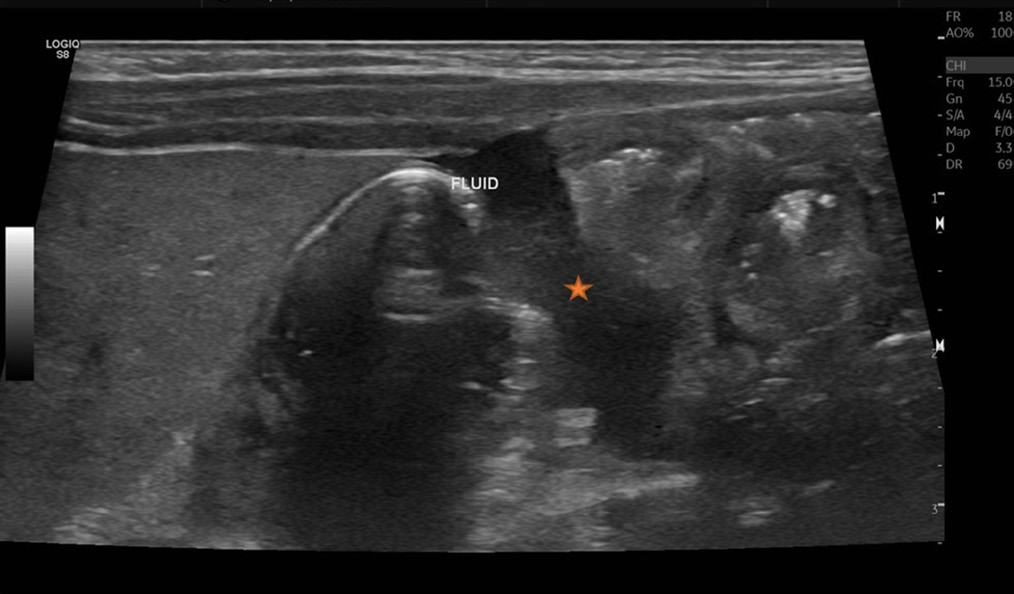
Mild free fluid also noted in abdomen (star).

## References

[1] Bryant AE, Laufer MR. Fetal ovarian cysts: incidence, diagnosis and management. J Reprod Med 2004; 49: 329–37. 15214704

[2] Crombleholme TM, Craigo SD, Garmel S, D'Alton ME. Fetal ovarian cyst decompression to prevent torsion. J Pediatr Surg 1997; 32: 1447–9 PMID. doi: 10.1016/S0022-3468(97)90558-3 9349765

[3] Kwak DW, Sohn YS, Kim SK, Kim IK, Park YW, Kim YH. Clinical experiences of fetal ovarian cyst: diagnosis and consequence. J Korean Med Sci 2006; 21: 690–4 PMIDPMCID. doi: 10.3346/jkms.2006.21.4.690 16891814 PMC2729892

[4] Nussbaum AR, Sanders RC, Hartman DS, Dudgeon DL, Parmley TH. Neonatal ovarian cysts: sonographic-pathologic correlation. Radiology 1988; 168, , (no. 3): 817–21. doi: 10.1148/radiology.168.3.3043551 3043551

[5] Trinh TW, Kennedy AM. Fetal ovarian cysts: review of imaging spectrum, differential diagnosis, management, and outcome. Radiographics 2015; 35: 621–35 PMID. doi: 10.1148/rg.352140073 25763743

[6] Galinier P, Carfagna L, Juricic M, Lemasson F, Moscovici J, Guitard J, et al. Fetal ovarian cysts management and ovarian prognosis: a report of 82 cases. J Pediatr Surg 2008; 43: 2004–9. doi: 10.1016/j.jpedsurg.2008.02.060 18970932

[7] Ngo A-V, Otjen JP, Parisi MT, Ferguson MR, Otto RK, Stanescu AL. Pediatric ovarian torsion: a pictorial review. Pediatr Radiol 2015; 45: 18451842–551844 quiz. doi: 10.1007/s00247-015-3385-x 26209957

[8] Oltmann SC, Fischer A, Barber R, Huang R, Hicks B, Garcia N. Cannot exclude torsion--a 15-year review. J Pediatr Surg 2009; 44: 1212–7. doi: 10.1016/j.jpedsurg.2009.02.028 19524743

[9] Słodki M, Respondek-Liberska M. Fetal ovarian cysts--420 cases from literature--metaanalysis 1984-2005. Ginekol Pol 2007; 78: 324–8. 17621997

[10] Amodio J, Hanano A, Hannao A, Rudman E, Banfro F, Garrow E. Complex left fetal ovarian cyst with subsequent autoamputation and migration into the right lower quadrant in a neonate: case report and review of the literature. J Ultrasound Med 2010; ; 29: 497–500 10.7863/jum.2010.29.3.497. Erratum in: J Ultrasound Med. 2010 May;29(5):866. Hannao, Amer [corrected to Hanano, Amer]. PMID: 20194948Mar. doi: 10.7863/jum.2010.29.3.497 20194948

[11] Enríquez G, Durán C, Torán N, Piqueras J, Gratacós E, Aso C, et al. Conservative versus surgical treatment for complex neonatal ovarian cysts: outcomes study. AJR Am J Roentgenol 2005; 185: 501–8 PMID. doi: 10.2214/ajr.185.2.01850501 16037528

[12] Pérez R et al. Congenital ovarian cyst: diagnosis and perinatal management. J Gynecol Neonatal Biol 2015; 1: 1–5.

